# High Proportion of Male Faeces in Jaguar Populations

**DOI:** 10.1371/journal.pone.0052923

**Published:** 2012-12-28

**Authors:** Francisco Palomares, Séverine Roques, Cuauhtémoc Chávez, Leandro Silveira, Claudia Keller, Rahel Sollmann, Denise Mello do Prado, Patricia Carignano Torres, Begoña Adrados, José Antonio Godoy, Anah Tereza de Almeida Jácomo, Natália Mundim Tôrres, Mariana Malzoni Furtado, José Vicente López-Bao

**Affiliations:** 1 Department of Conservation Biology, Estación Biologica de Doñana, CSIC, Sevilla, Spain; 2 Universidad Autónoma Metropolitana-Unidad Lerma, Lerma de Villada, México, México; 3 Jaguar Conservation Fund, Mineiros, Goias, Brazil; 4 Instituto Nacional de Pesquisas da Amazônia (INPA), Manaus, Amazonas, Brazil; 5 Department of Forestry and Environmental Resources, North Carolina State University, Raleigh, North Carolina, United States of America; 6 Department of Integrative Ecology, Estación Biológica de Doñana, CSIC, Sevilla, Spain; 7 Departament of Preventive Veterinarian Medicine and Animal Health, Faculdade de Medicina Veterinária e Zootecnia, Universidade de São Paulo, Sao Paulo, Sao Paulo, Brazil; Australian Wildlife Conservancy, Australia

## Abstract

Faeces provide relevant biological information which includes, with the application of genetic techniques, the sex and identity of individuals that defecated, thus providing potentially useful data on the behaviour and ecology of individuals, as well as the dynamics and structure of populations. This paper presents estimates of the sex ratio of different felid species (jaguar, *Panthera onca*; puma, *Puma concolor*; and ocelot/margay, *Leopardus pardalis/Leopardus wiedi*) as observed in field collected faeces, and proposes several hypotheses that could explain the strikingly high proportion of faeces from male jaguars. The proportion of male and female faeces was estimated using a non-invasive faecal sampling method in 14 study areas in Mexico and Brazil. Faecal samples were genetically analysed to identify the species, the sex and the individual (the latter only for samples identified as belonging to jaguars). Considering the three species, 72.6% of faeces (n = 493) were from males; however, there were significant differences among them, with the proportion from males being higher for jaguars than for pumas and ocelots/margays. A male-bias was consistently observed in all study areas for jaguar faeces, but not for the other species. For jaguars the trend was the same when considering the number of individuals identified (n = 68), with an average of 4.2±0.56 faeces per male and 2.0±0.36 per female. The observed faecal marking patterns might be related to the behaviour of female jaguars directed toward protecting litters from males, and in both male and female pumas, to prevent interspecific aggressions from male jaguars. The hypothesis that there are effectively more males than females in jaguar populations cannot be discarded, which could be due to the fact that females are territorial and males are not, or a tendency for males to disperse into suboptimal areas for the species.

## Introduction

For decades, faeces have provided relevant, and on occasion the only, biological information for many species [1]. With the application of genetic techniques, faeces not only allow species identification but may even provide information on the sex and identity of individuals [2]–[3], thus yielding potentially useful data on the behaviour and ecology of individuals, as well as the dynamics and composition of populations.

Using non-invasive faecal sampling and genetic analyses, several aspects of the ecology and genetic structure of jaguar populations were studied for three years in a number of study areas in Mexico and Brazil. During this study, a high proportion of male jaguar faeces was identified along with those of other felid species (pumas and ocelots/margays; see below) surveyed in the same study areas. The aim of this paper is to present these results and propose several hypotheses that could explain the strikingly high proportion of male jaguar faeces found.

The most intuitive idea to explain the higher male proportion of jaguar faeces is that male jaguars defecate more than females along the sites where faeces were collected, while the number of male and female jaguars in a population is similar. For example, some data suggest that this occurs in male Eurasian otters, Lutra lutra, and Eurasian badgers, Meles meles ([4]–[5] but see [6] for a different result). Felids, like many other carnivores, prefer to use clearly marked routes to move when available [7]–[8]–[9]. Thus, it is likely that individuals use these routes to deposit scent or visual marks (including faeces) in order to be able to communicate with other individuals [10]–[11]–[12]–[13]. Males may prefer to mark along such intensively used travel paths in order to demarcate their territories, or advertise their presence to other competing males or adult females [14]. In contrast, females might try to remain relatively unnoticed, because in the absence of a territorial male might have potential for infanticide [15]. If this is the case, we would expect to find more male faeces on the more heavily transited and conspicuous travel paths such as dirt roads, whereas in other less conspicuous and transited sites, the number of male and female faeces might be similar or higher for females.

Also, a higher proportion of male jaguar faeces might in part to be explained because there are more males present in jaguar populations. At least two different scenarios might explain a higher abundance of males than females in jaguars. First, if females are territorial and males are not, which appears to be the case [16]-[17]- [18] (but also see [19]), several males might be identified within a single female territory (in addition to the young from that female). Second, if males are the individuals that mainly disperse from natal areas, as it occurs in other felids [20]–[21], males would be the sex principally found in suboptimal areas (but see [14], for data suggesting that male and female jaguars may use suboptimal areas in a same way).

## Results

The species and the sex was determined for a total of 493 faeces (246, 216 and 31 from jaguars, pumas and ocelots/margays, respectively). Overall, 72.6% of faeces were from males, but significant differences were found among species (Chi-square test; *X^2^* = 31.635, *df* = 2, *P*<0.001; Table 1), with the proportion of faeces from males being higher for jaguars than for both pumas and ocelots/margays (*X^2^* = 21.734, *df* = 1, *P*<0.001 and *X^2^* = 18.489, *df* = 1, *P*<0.001, respectively). No significant difference was found between pumas and ocelots/margays (*X^2^* = 2.145, *df* = 1, *P* = 0.143).

**Table 1:** Number of faeces from male (M) and female (F) jaguars, pumas and ocelots/margays collected and identified for different study areas.

	Faeces						Individuals	
Study area	Jaguar		Puma		Ocelot/Margay		Jaguars	
	M	F	M	F	M	F	M	F
Calakmul	5 (100%)	0	4 (40%)	6	1	0	2 (100%)	0
20 Nov	0	0	1 (100%)	0	0	2		
Caoba	12 (75%)	4	5 (41.7%)	7	1	0	3 (75%)	1
El Edén	22 (95.7%)	1	13 (86.7%)	2	0	3	3 (75%)	1*
Zapotal	40 (100%)	0	2 (18.8%)	9	2	1	5 (100%)	0
Petcacab	10 (90.9%)	1	3 (33.3%)	6	0	0	3 (75%)	1
Ducke	15 (65.2%)	8	60 (95.2%)	3	1	0	3 (50.0%)	3
Uatumã	5 (62.5%)	3	8 (72.7%)	3	1	2	2 (66.7%)	1*
Viruá	7 (87.5%)	1	7 (53.8%)	6	4	3	2 (66.7%)	1*
Maracá	2 (100%)	0	4 (66.7%)	2	1	0	1 (100%)	0
Emas	14 (93.3%)	1	18 (56.3%)	14	–	–	3 (75%)	1*
Araguaia	–	–	1 (50%)	1	–	–		
Capivara	50 (76.9%)	15	5 (83.3%)	1	–	–	10 (68.8%)	7
Caiman	23 (76.7%)	7	8 (41.7%)	17	4	5	10 (71.4%)	4
Overall	204 (83.6%)	40	139 (64.7%)	76	15 (48.4%)	16	47 (71.2%)	19

Data were collected between 2004 and 2009 (values within brackets represent the percentage for males relative to the total number of faeces found in each study area and species). For jaguars, the number of different males and females identified is shown (values within brackets represent the percentage of males relative to the total number of individuals identified).

* No female faeces could be genotyped in these areas, but given that between one and three female faeces were found, one female was asigned to these areas.

Considering the study area as the sampling unit the mean proportion of male faeces was also higher than that of females for jaguars (Mann-Witney Rank Sum test; *T* = 222, *N* = 12, *P*<0.001), but not for pumas (*T* = 239.5, *N* = 14, *P* = 0.098) or ocelots/margays (*T* = 120, *N* = 10, *P* = 0.272). Furthermore, in all study areas more male than female faeces were found for jaguars, whereas for pumas or ocelots/margays in some areas more male faeces were found and in others more female faeces (Table 1).

For jaguars, the trend was the same when considering the number of different individuals identified (*N* = 66), with 71.2% of them being male (Table 1). There was a high correlation between the proportion of faeces from males and the number of different male individuals identified per area (*r_s_* = 0.866, *N* = 12, *P*<0.0001). We found on average 4.2±0.56 (*N* = 12 areas) faeces per male and 2.0±0.36 (*N* = 9 areas) faeces per female, the differences being statistically significant (*T* = 60.5, *P* = 0.007).

Although the observed proportion of male jaguar faeces was higher on dirt roads than on trails, and the observed proportion of female jaguar faeces was higher on trails than on dirt roads, however, probably due to low sample size for females, differences were not significant (*X^2^* = 1.925, *df* = 1, *P*<0.165; Table 2). For pumas, the proportion of male faeces and the proportion of female faeces was in both cases higher on trails than on dirt roads, but differences between sexes were also significant with a higher proportion in males than in females (*X^2^* = 6.798, *df* = 1, *P* = 0.009; Table 2). Nevertheless, when the data from one of the study areas (Ducke Reserve) was removed, differences between sexes were no longer significant (*X^2^* = 0.535, *df* = 1, *P*<0.535). We observed that 60 (62.5%) out of the 96 male puma faeces found on trails for all study areas were from Ducke Reserve, while only 8.8% of the 34 faeces from females were found in this same area. Thus results for males were greatly influenced by data from the Ducke Reserve where there were only trails and the faeces from male pumas clearly predominated. We did not obtain enough data to perform a similar analysis for ocelots/margays (Table 2).

**Table 2:** Number of faeces from male and female jaguars, pumas and ocelots/margays along dirt roads and animal or human trails.

	Jaguar		Puma		Ocelot/margay	
	Roads	Trails	Roads	Trails	Roads	Trails
Male	52 (53.6%)	45	9 (8.6%)	96	3	6
			9 (25.0%)*	36*		
Female	5 (31.3%)	11	12 (35.3%)	34	0	7
			12 (38.7%)*	31*		

Values within brackets represent the percentage of faeces along roads for each sex relative to the total number of faeces found for that sex.

* Number of faeces after removing data from the Ducke Reserve study area.

## Discussion

The proportion of male jaguar faeces is surprisingly high, even more so if the results are compared to those obtained for the other felid species living in the same study areas, where the proportion of male faeces was clearly lower and similar to that of females. The number of faeces found per male was higher than the number of faeces found per female. Fuerthermore, the number of different individual male jaguars identified was also higher than the number of different females detected and the male/female ratio was similar for faeces and individuals. These results would support the hypothesis that males defectate more than females along the routes (i.e. dirt roads and trails) where faeces were sampled. Results from camera traps, which normally are placed along similar routes to those where faeces were sampled in this study, also show that more males are photographed than females (see [22] for a review of studies). This further supports the idea that more male faeces are found as a result of a differential use between sexes of the routes sampled (see below). However, incorporating detectabilty analysis in future studies could help to definitively solve the question [23].

There is little information about the use of dirt roads and trails by male and female pumas and ocelot/margays. Our data suggest, mainly for pumas for which there are more data, that both sexes are likely to utilize these routes to a similar extent or that the use may vary locally as a function of area-specific variables. Howerver, the camera trapping information that exists indicates that male pumas, like jaguars, are more frequently photographed than females [9]–[24], and that in ocelots either a similar number of males and females or more females are normally photographed [25]–[26]–[27]–[7]. Nevertheless, information derived from camera traps are not entirely comparable to that obtained from faeces given that in camera trapping studies some doubt always remains about sex-ratios as males are easier to distinguish than females.

We tested whether males prefer more than females to deposite faeces on the most conspicuous routes by separating data obtained from faeces collected on well-established dirt roads (i.e. traversable by car), and on animal and human trails, under the assumption that the former are preferred as travelling paths and marking sites [9]. Results were not conclusive for this last proposition. For jaguars, although the proportion of male faeces was higher on dirt roads and that of females was higher on trails, sample size probably prevented the detection of significant differences. Salom-Pérez et al. [28] suggest that females avoid travelling on dirt roads as two out of the three females detected in their study roamed off-road, and for general habitat models female jaguars were found to avoid roads more often than males [29].

Considering pumas, the proportion of both male and female faeces was higher on trails. If the spacing patterns of pumas in the neotropics is similar to that described in northern populations, where males are territorial and females are not [21], the potential for infanticide is lower than for jaguars and both sexes might use dirt roads and trails in a similar manner. That would still not explain why pumas, as compared to jaguars, defecate more on trails than on dirt roads. It is possible that pumas try to prevent interspecific aggressions from male jaguars, who seem to prefer to move along dirt roads. Some studies have already suggested avoidance between pumas and jaguars [30]–[31]–[18]–[32] and considering its larger size, the jaguar is generally assumed to be the competitively dominant species.

It seems clear that male jaguars are using the routes sampled more frequently and that they deposit more faeces there than females. However, the question still remains whether there are more males present in jaguar populations as well, which might in part explain the pattern found. We discard the possibility that male jaguars produce more faeces than females, which occurs in the oribi (*Ourebia ourebi*), where males reduce depositions in order to defecate more than females in some parts of their home ranges [33]. Physiological characteristics of carnivores are markedly different from those of ungulates. Furthermore, the number of faeces produced by males and females is similar in captive pumas (O. Monroy-Vilchis, pers. comm.).

As stated in the introduction at least two different scenarios might explain a higher abundance of males than females in jaguars: 1) that females are territorial and males are not [16]–[17]– [18] (but also see [19]), and 2) that males are the individuals that mainly disperse from natal areas [20]–[21] (but see [14]). Under the first hypothesis, and assuming similar marking rates with faeces between the sexes, a couple of predictions can be made: (a) more faeces from males than from females should be found, and (b) in the case of finding other female faeces, they should belong to closely related individuals (i.e. daughters, siblings). Looking at the data presented in Table 1, two or three males were found in six different areas where only a single female was detected, which supports the first hypothesis. With data obtained by radio-tracking, Cavalcanti and Gese [17] also found at least 2–3 males within female home ranges. Note, however, that to find more male faeces, home ranges of males should be of similar size to those of females. If male home ranges are larger than those of females, the number of male faeces would depend on the size difference between male and female home ranges. For example, if the size of male home ranges is twice that of females the number of faeces from males would be twice that of females, and if male home range size is four times that of females, a similar number of faeces from males and females should be found. Although most studies thus far conducted rely on small sample sizes, reported home ranges of female jaguars tend to be smaller than those of males [34]– [16]–[35]. For example, in the Pantanal (Brazil) males had home ranges between 2.3 and 2.7 times larger (depending on the season and home range estimator) than females [17], and home ranges of males were between 2.7 and 4.2 times larger in the Yucatan (México; [36]). While we cannot discard the hypothesis that there are more male than female jaguars in some of the populations sampled in the present study, this is most likely not the case of one of them, in Emas National Park, where Sollmann et al. [37] estimated a sex ratio skewed toward females based on camera trap data. In fact, a female-biased sex ratio has been reported for several large felid species including the jaguar [38]–[39]. Unfortunately, our data were insufficient to perform a test of the second prediction, but in other solitary felids such as bobcats (*Lynx rufus)* females were more closely related than males [20].

The spatial pattern in pumas seems to differ from that of jaguars, with males being territorial and females exhibiting weak territoriality [21]. According to this pattern, it would be expected that more female than male faeces would be found, but this was not the case. An explanation for this conflicting result might be that the spatial organization of pumas in the sampled areas is different to that described for northern populations [21]. The populations considered in this study live in different habitats and eat different prey, and thus pumas in these populations might have a different spatial organization. Unfortunately there is no data on the subject for puma populations in Latin America. However, the number of puma faeces from males and females should also be affected by differences in home range size between the sexes, as stated above for jaguars. Limited data indicate that in the neotropics male pumas might have larger home ranges than females [30]–[40]–[41].

According to the second scenario (i.e. males are using suboptimal areas), it would be expected that when suboptimal areas for jaguars are surveyed, the turnover rates of male jaguars in these areas would be higher than in other areas where female jaguars are consistently found (i.e. in optimal areas for jaguars). A similar argument would apply to pumas, since males are also the individuals that mainly disperse [21]. However, compared to jaguars, pumas are more generalist in habitat and prey use [30]–[35]–[42]–[43]. Therefore, suboptimal areas for jaguars might be optimal for pumas. Thus, if mainly male pumas are found in an area where male and female jaguars are found as well (i.e. these areas would not be suboptimal for pumas), the biased sex-ratio toward males might be a consequence of direct competition with jaguars [31] rather than of habitat quality.

## Materials and Methods

### Ethics Statement

We thank the ICMBio for granting permission to work at the Caiman Ecological Refuge (Permit no. 11214) and Serra da Capivara National Park (Permit no. 13781). Sampling in the Brazilian Amazon was carried out under licenses n° 131/2005 CGFAU/LIC, 13883-1 SISBIO and 15664-1 SISBIO of the Instituto Brasileiro do Meio Ambiente – IBAMA, and in the Mexican areas under the licence SGPA/DGVS/549 provided by Martín Vargas of the Dirección General de Vida Silvestre (Semarnat). Faecal samples were exported from Brazil to Spain for genetic analysis under IBAMA/CGEN Autorização de Acesso licence n° 063/05 and IBAMA/CITES export licences n° 0123242BR, 08BR002056/DF and 09BR003006/DF, and from Mexico to Spain under the export licences n° MX33790 and MX42916 of the Secertaria de Medio Ambiente/CITES. All the study species here are protected. Nevertheless, we did not manage them and only collected their feces (see below).

### Study Areas

**Figure 1:**
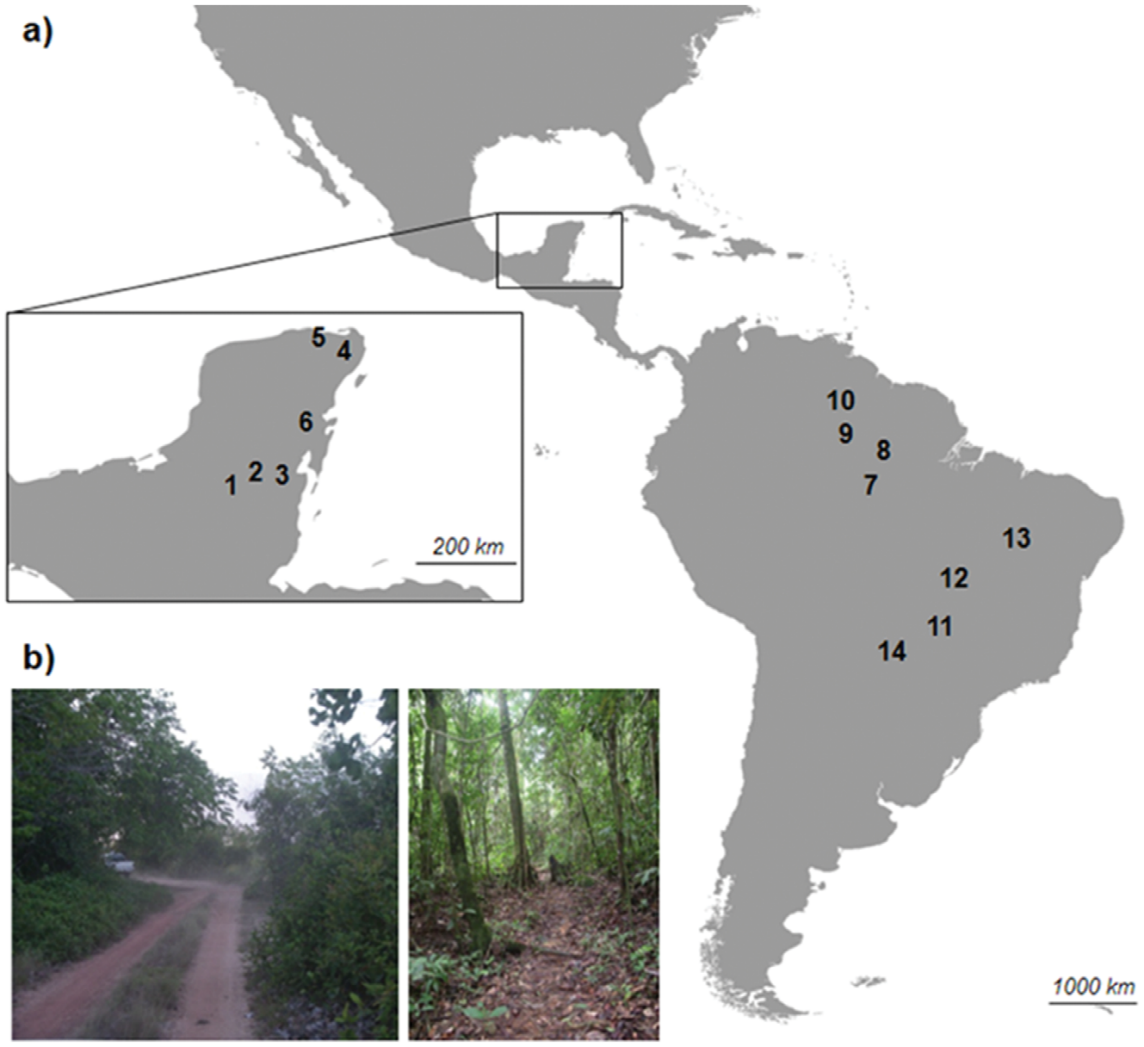
Location of the study areas and type of ways sampled. a) Map showing the approximate location of the 14 study areas (Mexico: 1) Calakmul, 2) Ejido 20 Noviembre, 3) Caoba, 4) Edén, 5) Zapotal, 6) Petcacab, and Brazil: 7) Ducke, 8) Uatumã, 9) Viruá, 10) Maracá, 11) Emas, 12) Araguaia, 13) Capivara and 14) Caima). b) Pictures showing a dirt road (left) and a trail (right).

Fecal samples were collected in six areas from the Yucatan Peninsula, Mexico (Calakmul, 18° 11′ 05″ N, 89° 44′ 49″ W; El Eden, 21° 13′ N, 87° 11′ W; Zapotal, 21° 20′ 25″ N, 87° 36′ 20″ W; Ejido Caoba, 18° 14′ N, 89° 03′ W; Ejido 20 Noviembre, 18° 25′ 35″ N, 89° 18′ 12″ W; and Ejido Petcacab, 19° 17′ 15″ N, 88° 13′ 33″ W), four in the Amazon, Brazil (Ducke Reserve, 02° 55′ S, 59° 59′ W; Uatumã Biological Reserve, 1° 46′ S, −59° 16′ W; Viruá National Park, 1° 29′ 9″ N, 61° 2′10″ W; and Maracá Ecological Station, 3° 24′ 26″ N, 61° 29′ 13″ W), one in the Pantanal, Brazil (Refúgio Ecológico Caiman, 19° 57′ S, 56° 18′ W), two in the Cerrado, Brazil (Emas National Park, 18° 19′ S, 52° 45′ W; Araguaia River, between latitudes 3° 25′ 13″ and 180° 15′ 40″ S and longitudes 53° 26′ 26″ and 47° 53′ 07″ W), and one in the Caatinga, Brazil (Serra da Capivara National Park, 8° 26′ S, 42° 19′ W) (Fig. 1a).

The Yucatan peninsula is characterized by tropical rain forest and semi-evergreen forest, and to a lesser extent tropical deciduous forest and seasonally flooded forest. The Ejido 20 Noviembre, Ejido Caoba y Ejido Petcacab are characterized by mixed tropical rain forest and semi-evergreen forest. Calakmul is a semi-evergreen forest and seasonally flooded forest, whereas Zapotal and El Edén present a mix of semi-deciduous tropical forest and secondary vegetation, seasonally flooded forest, savannas and aquatic vegetation.

From the Amazon basin, Ducke and Uatumã are covered by wet tropical rainforest, while Viruá and Maracá are characterized by mosaics of vegetation formed by transitions between savannas and tropical upland forest. The Refúgio Ecológico Caiman is a cattle ranch and ecotourism business located in the Pantanal wetlands and it is characterized by a mosaic of floodplains, grasslands, savannas, riparian forests and exotic pastures. Emas National Park is situated in the Cerrado grasslands, where large tracts of grassland plains are interspersed with patches of shrub fields, marshes, and riparian forest. It is virtually surrounded by crop plantations. The Araguaia River originates in the Cerrado and flows northward into the Amazon biome. The Serra da Capivara National Park is situated in the semi-arid Caatinga biome and it is predominantly covered by a 6–10 m tall shrubby vegetation.

### Collection of Faeces

Most faeces were sistematically collected by slowly walking once dirt roads and animal- and human-made trails. Some faeces were opportunistically collected while doing other research activities or moving through the study areas. Except in rare occasions, faeces were collected during the dry season. Areas were once sampled between 2007 and 2009, except in Ducke Reserve, which was also sampled in 2004 and 2005, and Maracá, Uatumã and Viruá that were sampled twice with one year between samplings. In the study areas of Caiman, Serra Capivara, Emas National Park and Araguaia River faeces were collected with the help of scat detector dogs [44]–[45]. The dogs were trained to detect jaguar and puma faeces, so that in areas where they were used mainly jaguar and puma faeces were obtained. However, in the other areas, all faeces that seemed to be from felids were collected. Genetic species identification confirmed that we collected jaguar, puma, and ocelot/margay faeces. With the genetic markers used, it was not possible to distinguish between the latter two species [46]. Considering that ocelots are more abundant and widespread [47], we suppose that most of these faeces were from this species.

The location of faeces was georeferenced with the aid of a GPS. For fresh samples, a small portion was inititally stored in 96% ethanol for 24–48 hours and then transferred to silica gel for storage; dried samples were directly stored in silica until genetic analyses were conducted.

### Genetic Analysis

DNA was extracted from faecal samples using protocols based on the GuSCN/silica method [48]–[49]–[50] and further purified and concentrated through ultrafiltration using Microcon-30 (Millipore). Species identification was performed using species-specific primers previously developed [46].

Sex identification was based on a method previously described by Pilgrim et al. [3] and optimized in this study for its use in faecal samples for the jaguar and other American felid species such as puma, ocelot and margay [51]. This method is based on the size difference between the male and female Amelogenin gene in chromosome Y (AMELY). Primer pairs were redesigned to exclude human gene amplification. Optimization of the newly designed primers (FRedi: 5′-TCAAGATGTTTCTCAGTCC-3′, RX: 5′-CTTTGTGCCTTACCATGCAG-3′ and R2Y-5′- CCCCCTGAGGGATAGTTTGT-3′) was carried out for both a range of temperatures (from 53 to 60°C) and template DNA quantities (10 pg–50 ng) to evaluate the robustness of the amplification from low quality DNA, and especially to evaluate the likelihood of ‘false females’ due to failed amplification of the shorter male-specific product. Amplifications were performed four times along with one male positive control DNA, one female positive control DNA and one negative control. PCR reactions consisted of 4 µl of DNA extract in a final volume of 20 µl, containing 67 mM Tris-HCl pH 8, 16 mM (NH4)2SO4, 2 mM MgCl2, 0,25 mM dNTPs, 0,8 mg/ml BSA, 0.6 µM of each primer and 0,4 U of Taq polymerase (Bioline). Cycling steps included a first denaturation step at 94°C for 2 minutes, followed by 40 cycles of denaturation at 92°C, annealing at 58°C and extension at 72°C, each step lasting 30 seconds, and a final extension step of 5 min at 72°C. The PCR products were run on 2% agarose gels. Females were scored whenever the upper band was seen at least three times with no amplification of the lower male-specific band, while for males only two independent amplifications of the male-diagnostic band were required.

For individual genotyping, an optimized set of 11 domestic cat microsatellite markers was used (Fca024, Fca126, F115a, Fca176, Fca026, Fca082b, Fca077, Fca090, Fca043, Fca547b, Fca566b; [52]), as described more extensively in Roques et al. [51].

### Data Analysis

The proportion of faeces coming from males and females of each species was calculated for the whole set of samples and for every study area. When possible the sampling unit was the study area, but for some analyses the whole set of data was used. When more than one survey was carried out in a given study area, samples were pooled for analysis. We adopted this approach because 1) normally the number of faeces identified and sexed in any given survey was small for most study areas, and 2) in some cases the same individual jaguars (the only species for which we had individual identification) were found in different years.

We compared the proportion of faeces from males and females found on dirt roads (i.e. man-made, clearly visible and accessible by car) and animal or human trails (i.e. often less visible and not accessible by car), under the assumption that the first ones are more conspicuous than the second ones (Fig. 1b).

We also calculated the number of faeces found and identified per male and female jaguar by dividing the total number of faeces identified for each sex by the number of different individuals identified of each sex in a given study area.
